# Recurrent small variants in *NESP55*/*NESPAS* associated with broad *GNAS* methylation defects and pseudohypoparathyroidism type 1B

**DOI:** 10.1172/jci.insight.185874

**Published:** 2024-12-20

**Authors:** Dong Li, Suzanne Jan de Beur, Cuiping Hou, Maura R.Z. Ruzhnikov, Hilary Seeley, Garry R. Cutting, Molly B. Sheridan, Michael A. Levine

**Affiliations:** 1 Center for Applied Genomics, and; 2 Division of Human Genetics, The Children’s Hospital of Philadelphia, Philadelphia, Pennsylvania, USA.; 3 Department of Pediatrics, University of Pennsylvania Perelman School of Medicine, Philadelphia, Pennsylvania, USA.; 4 Division of Endocrinology and Metabolism, University of Virginia School of Medicine, Charlottesville, Virginia, USA.; 5 Neurology and Neurological Sciences, Pediatrics, Division of Medical Genetics, and; 6 Division of Pediatric Endocrinology, Stanford University and Lucile Packard Children’s Hospital, Palo Alto, California, USA.; 7 Department of Genetic Medicine, Johns Hopkins University School of Medicine, Baltimore, Maryland, USA.; 8 Division of Endocrinology and Diabetes and The Center for Bone Health, The Children’s Hospital of Philadelphia, and Department of Pediatrics University of Pennsylvania Perelman School of Medicine, Philadelphia, Pennsylvania, USA.

**Keywords:** Endocrinology, Genetics, Bone disease, Genetic diseases, Molecular diagnosis

## Abstract

Pseudohypoparathyroidism type 1B (PHP1B) is associated with epigenetic changes in the maternal allele of the imprinted *GNAS* gene that inhibit expression of the α subunit of G_s_ (G_s_α), thereby leading to parathyroid hormone resistance in renal proximal tubule cells where expression of G_s_α from the paternal *GNAS* allele is normally silent. Although all patients with PHP1B show loss of methylation for the exon A/B differentially methylated region (DMR), some patients with autosomal dominant PHP1B (AD-PHP1B) and most patients with sporadic PHP1B have additional methylation defects that affect the DMRs corresponding to exons *XL*, *AS1*, and *NESP*. Because the genetic defect is unknown in most of these patients, we sought to identify the underlying genetic basis for AD-PHP1B in 2 multigenerational families with broad *GNAS* methylation defects and negative clinical exomes. Genome sequencing identified small *GNAS* variants in each family that were also present in unrelated individuals with PHP1B in a replication cohort. Maternal transmission of one *GNAS* microdeletion showed reduced penetrance in some unaffected patients. Expression of AS transcripts was increased, and NESP was decreased, in cells from affected patients. These results suggest that the small deletion activated AS transcription, leading to methylation of the *NESP* DMR with consequent inhibition of NESP transcription, and thereby provide a potential mechanism for PHP1B.

## Abstract 2

We identified two novel, small, recurrent variants in the *GNAS* gene in patients with autosomal dominant and sporadic pseudohypoparathyroidism type 1B that are associated with broad defects in methylation of the *GNAS* locus.

## Introduction

**Figure 1:**
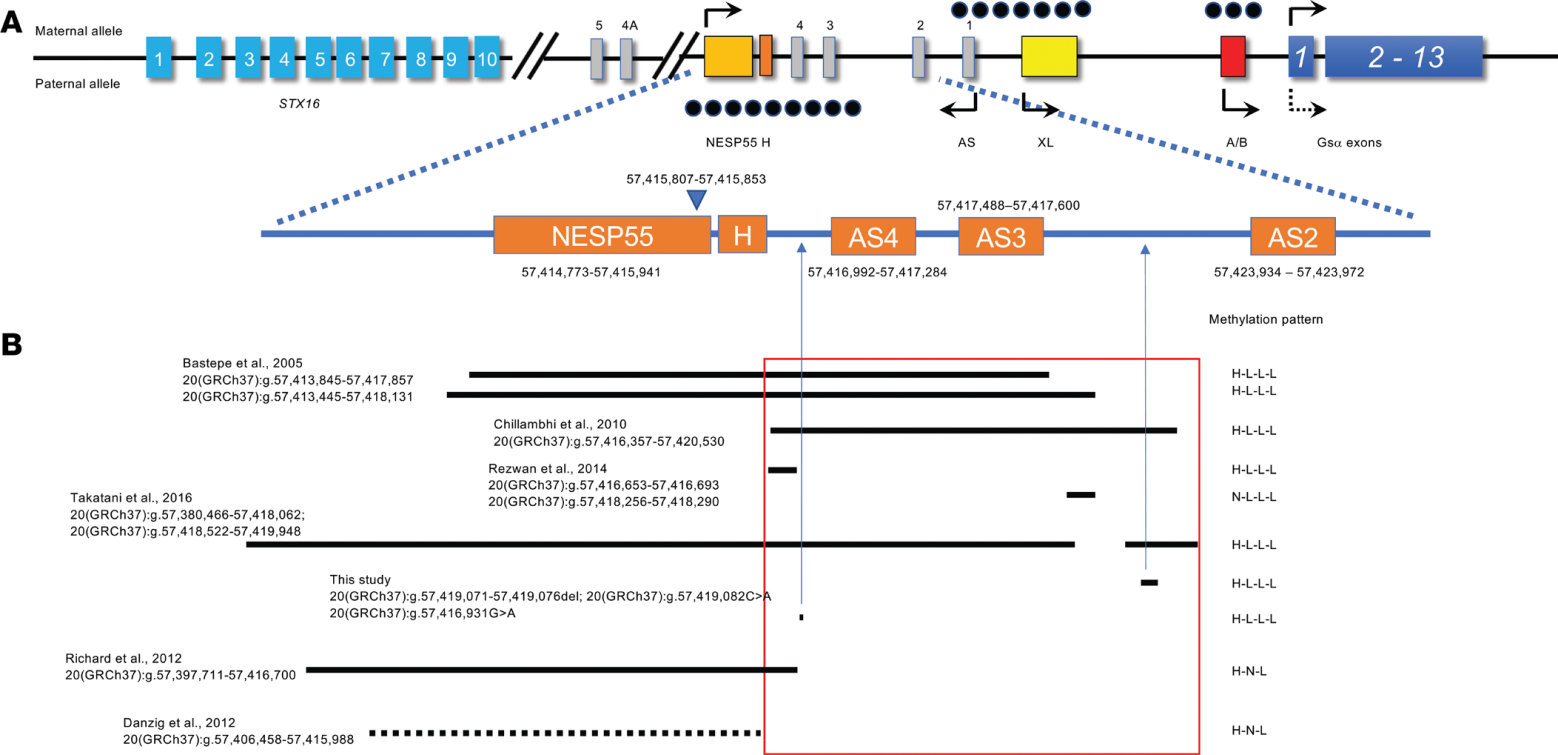
Schematic depiction of the region from the *STX16* gene to the *GNAS* complex locus and variants identified in patients with AD-PHP1B. (**A**) Schematically depicted locations of *STX16*, *GNAS*, *AS*, and putative GNAS ICRs. Boxes represent exons of *STX16*, *GNAS*, and *AS*, and filled black dots represent putative GNAS ICRs. (**B**) Distribution of microdeletions in patients with AD-PHP1B overlapping the NESP/AS region and 2 variants identified in this study. The deletion reported in Danzig et al. (28), presented as a dashed line, was confirmed via genome sequencing but was not specified in the original publication.

Pseudohypoparathyroidism type 1B (PHP1B; also termed inactivating PTH/PTHrP signaling disorder, iPPSD; ref. 1) is a disorder of hormone resistance in which the inability of parathyroid hormone (PTH) to activate endocrine signaling processes in target cells leads to a state of functional hypoparathyroidism (2–4). The pathophysiology of PHP1B is the result of genetic or epigenetic defects in the *GNAS* gene that reduce expression or function of the α subunit of the heterotrimeric stimulatory G protein (G_s_α), a signaling protein that couples heptahelical receptors to activation of adenylyl cyclase (4). *GNAS* is a complex imprinted gene on chromosome 20q13.3 (5) that utilizes multiple alternative promoters and first exons to generate various transcripts based on the parent of origin of each allele (6–8) (Figure 1A). G_s_α is the principal product of *GNAS* and is encoded by exons 1–13; G_s_α is expressed from both parental alleles in most tissues, but in some cells such as proximal renal tubular epithelium, pituitary somatotrophs, gonads, thyroid epithelial cells, and regions of the central nervous system, transcription of G_s_α occurs predominately from the maternal *GNAS* allele (9–17). The mechanism that accounts for allelic silencing of paternal G_s_α in some tissues is uncertain, but restricted expression of additional *GNAS* transcripts is achieved by use of promoters that are in differentially methylated regions (DMRs) (Figure 1A). Transcription of *NESP* from the paternal *GNAS* allele is inhibited by methylation of CpG dinucleotides within the *NESP* DMR (*GNAS-NESP*:TSS-DMR), whereas generation of transcripts from exons *XL*, *AS1*, and *A/B* (termed exon 1A in mice) is inhibited from the maternal *GNAS* allele by methylation of the corresponding DMRs (*GNAS-AS1*:TSS-DMR, *GNAS-XL*:Ex1-DMR, and *GNAS A/B*:TSS-DMR) on that allele (7). In addition, a fifth DMR, termed *GNAS-AS2*:TSS-DMR (18) and which appears to consist of 2 subdomains (19), has recently been identified and is located telomeric of *GNAS-AS1*:TSS-DMR.

Imprinting of *GNAS* leads to variable phenotypes based on the parental origin of the defective allele. Mutations that affect exons 1–13 of *GNAS* and directly reduce expression or function of G_s_α are the basis for tissue resistance to multiple hormones plus features of Albright hereditary osteodystrophy (AHO) in patients with PHP1A (OMIM 103580, also termed iPPSD type 2; ref. 2) when they are on the maternal allele and for pseudopseudohypoparathyroidism (PPHP; OMIM 612463), characterized by AHO only, when they lie on the paternal allele (20, 21). By contrast, PHP1B (OMIM 603233; also termed iPPSD3) is associated with characteristic epigenetic changes in the maternal *GNAS* allele that lead to reduced expression of G_s_α. Clinically, this manifests most notably in the renal proximal tubule and thyroid follicular cells, and results in renal resistance to PTH and sometimes resistance to thyroid-stimulating hormone (TSH) (2–4, 19). To date, all patients with PHP1B have loss of methylation (LOM) of the maternal DMR for exon A/B. LOM can be restricted to exon A/B, as occurs in most familial forms of PHP1B (autosomal dominant PHP1B [AD-PHP1B]). Alternatively, A/B DMR LOM can be associated with broader methylation defects that include LOM at all 3 maternal DMRs (*XL*, *AS1*, and *A/B*) and gain of methylation (GOM) at the paternal DMR associated with *NESP* (22–25), as found in rare families (see below) or most frequently in patients with sporadic PHP1B (80%–85% of PHP1B patients). Although in some cases partial (23–25) or complete (4, 22, 23, 26, 27) paternal uniparental disomy (patUPD) for chromosome 20 has been found, the genetic basis for most cases of sporadic PHP1B remains unresolved (19, 28) and has been proposed to result from either a failure in imprint establishment during oogenesis (29) or a lack of imprint maintenance after fertilization (28).

Approximately 10% of patients with PHP1B show autosomal dominant transmission of the disorder that is associated with epigenetic defects on the maternally inherited *GNAS* allele. In most cases, this is associated with a methylation defect that is limited to LOM of the exon *A/B* DMR and is caused by overlapping heterozygous microdeletions within the maternal *STX16* allele approximately 170 kb centromeric of the *GNAS* locus that include exon 4 and the adjacent portion of intron 4 (28, 30, 31). Exon 4 of *STX16* presumably represents an imprint control region (STX-ICR) that exerts long-range *cis*-regulatory control of an ICR located in the NESP55 exon (NESP-ICR) that is required for methylation and transcriptional silencing of the *GNAS* exon A/B (32). In addition, a large inversion located 7,225 bp downstream of *GNAS* exon XL (33) and retrotransposon insertions located approximately 1,200 bp telomeric of *GNAS* exon XL have also been identified as causes of altered methylation that is limited to *GNAS* exon A/B (34, 35). By contrast, a small number of patients with AD-PHP1B have broad methylation defects with GOM at DMRs for NESP55, and LOM at the XL, AS1, and exon A/B. In most cases, this is due to heterozygous deletions affecting the NESP55 and/or AS4/AS3 exons on the maternal *GNAS* allele (28, 36–39) within a region that represents a second ICR (NESP-ICR) (Figure 1A) (32). Finally, broad methylation defects in some patients with AD-PHP1B have been associated with rare duplications or complex rearrangements that involve DMRs in the alternative first exon(s) (40, 41). Nevertheless, apart from these rare defects, the majority of patients with PHP1B, both familial and sporadic, with broad methylation defects at the *GNAS* cluster do not have an obvious genetic alteration. Here, we describe the identification of 2 previously unidentified and recurrent small variants within the NESP-ICR of *GNAS* in individuals with familial and sporadic PHP1B who have global methylation defects. Our findings suggest that these deletions activate expression of the maternal *GNAS* antisense transcript and thereby convert the maternal *GNAS* allele to a paternal epigenotype.

## Results

### Family 1.

**Figure 2:**
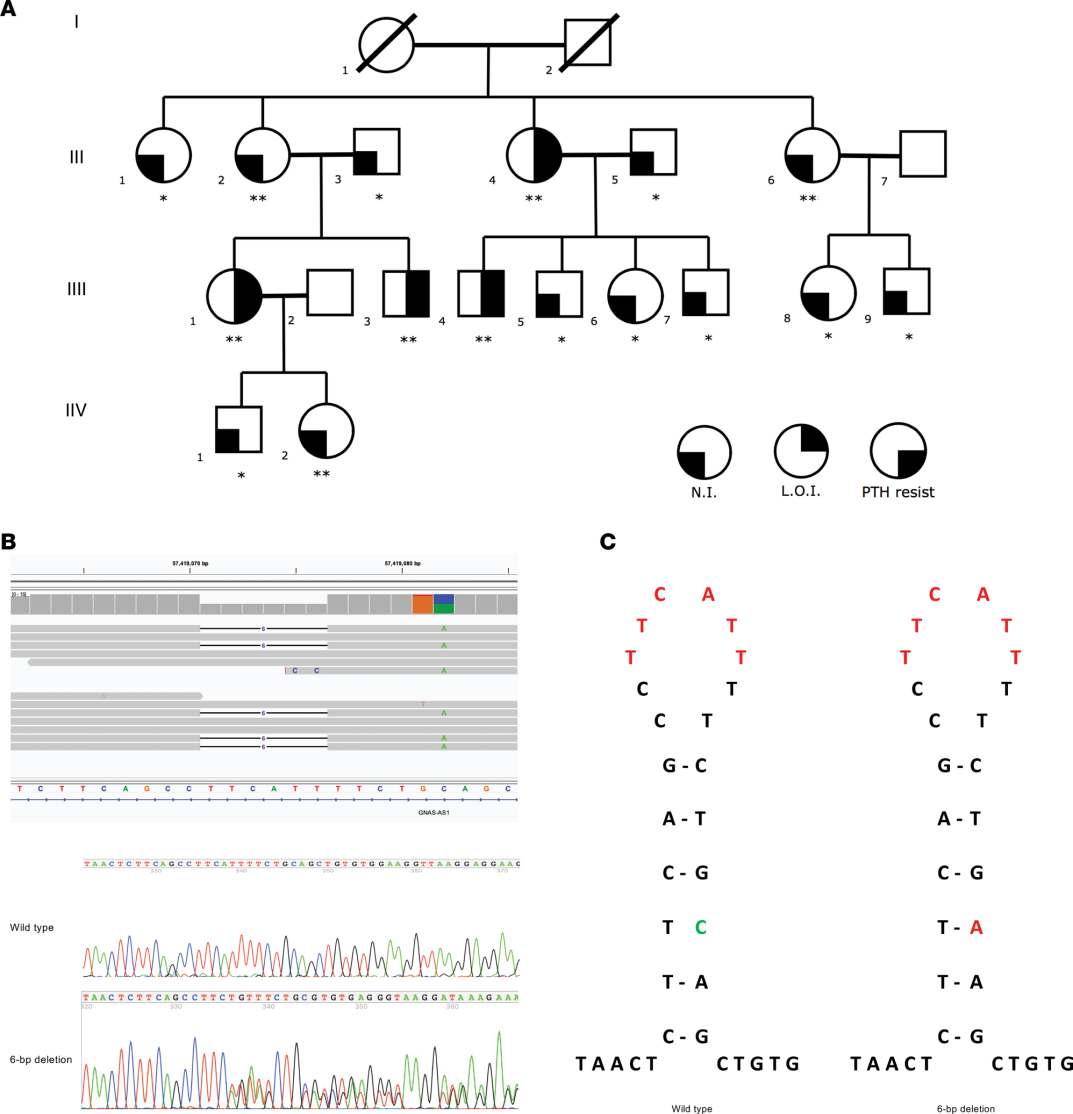
Pedigree of family 1, identified 6-bp NESP55/NESPAS intronic deletion, and proposed mechanism of deletion formation. (**A**) Pedigree of family 1 with AD-PHP1B. *Tested negative for the NESP55/NESPAS intronic variant (20[GRCh37]:g.57419071–57419076delTTCATT) associated with a nearby single-nucleotide transversion (20[GRCh37]:g.57419082C>A) in *cis*. **Tested positive for the NESP55/NESPAS intronic variant. (**B**) Sanger sequencing showed the presence of the heterozygous NESP55/NESPAS intronic variant. (**C**) Proposed stem-loop structure with 1 mismatch to explain the variant formation. N.I., normal imprinting (methylation); L.O.I., loss of imprinting (abnormal methylation); PTH resist, resistance to parathyroid hormone.

We performed genome sequencing (GS) on DNA samples from affected individuals III-1 and III-4 and confirmed normal sequences for *GNAS* exons 1–13 and the intervening introns. Due to our prior demonstration of linkage between the *GNAS* locus and AD-PHP1B in this family (42, 43), we systematically analyzed the 20q13.3 locus, where *GNAS* is located, for structural variations (SVs). Despite using multiple in silico algorithms (i.e., BreakDancer, Manta, Wham, CNVnator, and Lumpy), we were unable to identify an SV in either of the 2 affected patients. Next, we scrutinized the *GNAS* locus for relatively smaller insertions/deletions (indels) and duplications (i.e., <50 bp), which disclosed a heterozygous 6-bp deletion (20[GRCh37]:g.57419071–57419076delTTCATT) associated with a nearby single-nucleotide transversion (20[GRCh37]:g.57419082C>A) in *cis* in these 2 patients (Figure 2, A and B). The 2 variants are on the same allele, which was also confirmed by subsequent Sanger sequencing in the entire family (hereafter we refer to this defect as the 6-bp deletion). The 6-bp deletion was intronic to both NESP55 and AS1 — in the first intronic region of the NESP55 transcript and the second intronic region of the antisense AS1 transcript (Figure 1). The variant was absent in 1000 Genomes Project, gnomAD v2 with 15,708 genomes, gnomAD v4 with 76,215 genomes, and additional genome-sequencing data from over 10,000 samples in the Human Longevity database. Furthermore, we analyzed nucleotide sequences of the deletion and surrounding 20 bp. It is noteworthy that there are 6-bp inverted repeat sequences flanking the deletion that could form a small stem-loop structure with one mismatch (Figure 2C, green marked). The 6-bp deleted sequence (20[GRCh37]:g.57419071–57419076delTTCATT) resides at the loop of the putative structure (Figure 2C, red highlighted) and the base change (20[GRCh37]:g.57419082C>A) at the stem of the structure could have occurred during DNA replication in the context of slipped-strand mispairing (44).

In addition to III-1 and III-4, the other 2 affected members of this family (proband II-4 and III-3) also carried the variant (Figure 2A). All affected members of generation III had inherited the variant from their mother and had broad defects in methylation. DNA was not available from individuals I-1 and I-2, however, so it was not possible to identify the parent of origin of this defect.

Two of the proband’s unaffected sisters (II-2 and II-6), an unaffected great niece (IV-2), and an unaffected great nephew (IV-1) had inherited the mutation on a maternal *GNAS* allele, but had completely normal methylation patterns and lacked evidence of PHP1B, consistent with our original proposal that there is incomplete penetrance of this *GNAS* defect (42, 45). These observations, plus the absence of any other genetic variations in the *GNAS* locus, provide additional evidence that the 6-bp defect is associated with AD-PHP1B in affected members of this family.

### Family 2.

**Figure 3:**
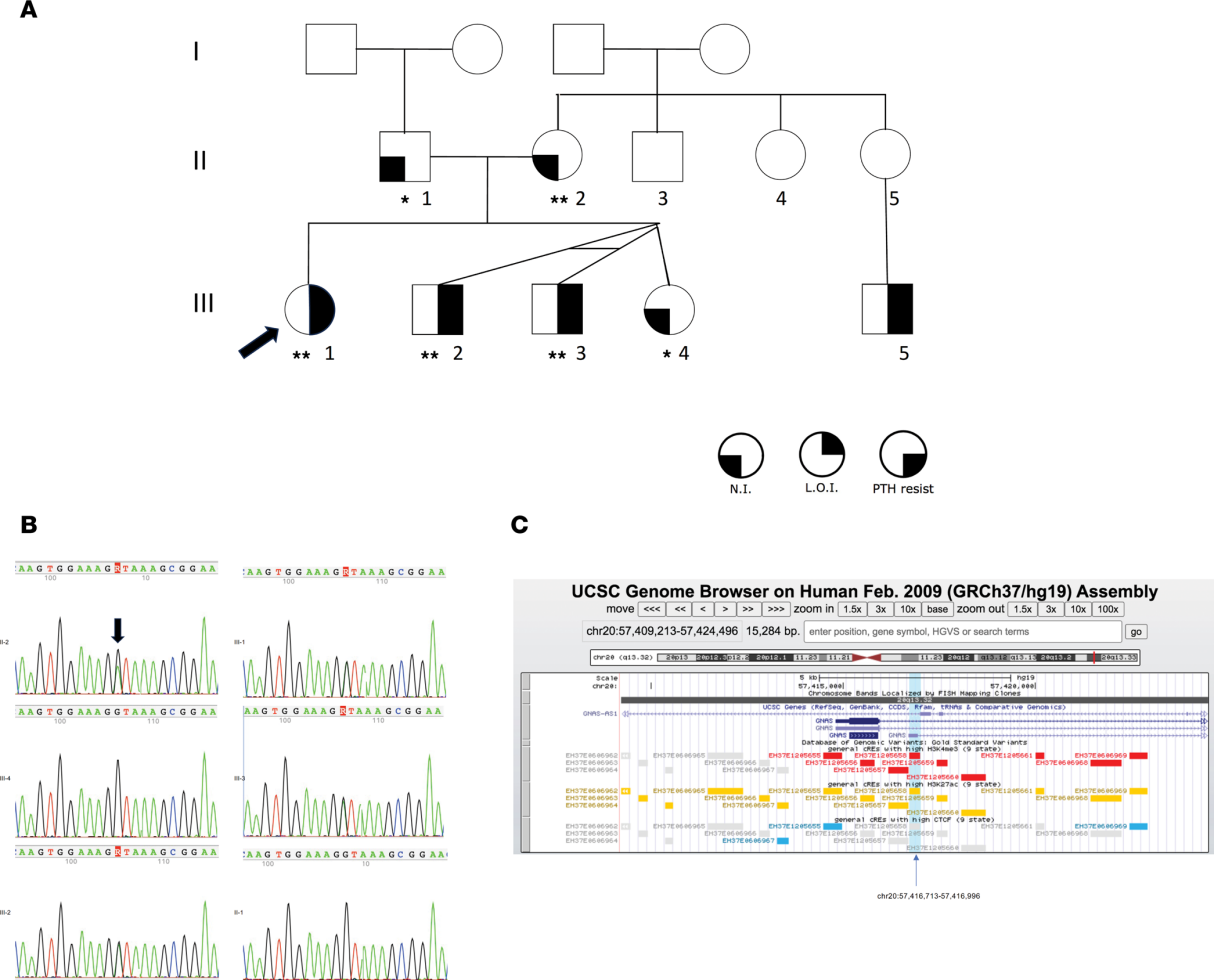
Pedigree of family 2 and identified intronic point variant. (**A**) Pedigree of family 2 with AD-PHP1B. *Tested negative for the point intronic variant. **Tested positive for the point intronic variant. N.I., normal imprinting (methylation); L.O.I., loss of imprinting (abnormal methylation); PTH resist, resistance to parathyroid hormone. (**B**) Sanger sequencing in available family members of family 2. (**C**) A UCSC screenshot showing the intronic point variant overlapping with a *cis*-regulatory element (ccRE) annotated by ENCODE (accession number EH37E1205658).

The molecular basis for PHP1B in kindred 2 (Figure 3A) was not disclosed by previous exome sequencing. We performed GS on DNA samples from all 6 available family members and identified a point variant (20[GRCh37]:g.57,416,931G>A; Figure 3B) in the first intronic region of the NESP55 transcript and near the donor splice site of AS1 exon 4 (NR_002785.2: n.819+61C>T; Figure 1) in all 3 affected individuals with broad methylation defects (III-1, III-2, and III-3) and their unaffected mother (II-2) with normal methylation of DMRs, consistent with maternal transmission of PHP1B. DNA samples were not available from the parents of II-2, who presumably carries the variant on a paternal *GNAS* allele. Both the unaffected father (II-1) and unaffected sister (III-4) had wild-type *GNAS* alleles and normal methylation patterns (see below).

This variant was not present in the 1000 Genomes Project, gnomAD datasets (v2 and v4), and the Human Longevity database. To further annotate the variant, we queried ENCODE data and found the variant is in a *cis*-regulatory element (ccRE; ENCODE accession number EH37E1205658) with the *GNAS* gene nearby. This ccRE has maximum DNase, H3K4me3, H3K27ac, and CTCF *z* scores of 2.25, 4.99, 2.75, and 1.48, respectively, highly suggested it has a promotor-like property regulating *GNAS* expression (Figure 3C). However, since ENCODE data were primarily developed to annotate the relationship between ccREs and protein-coding gene expression, this ccRE’s effect on noncoding gene expression is unknown.

### Replication cohort.

**Figure 4:**
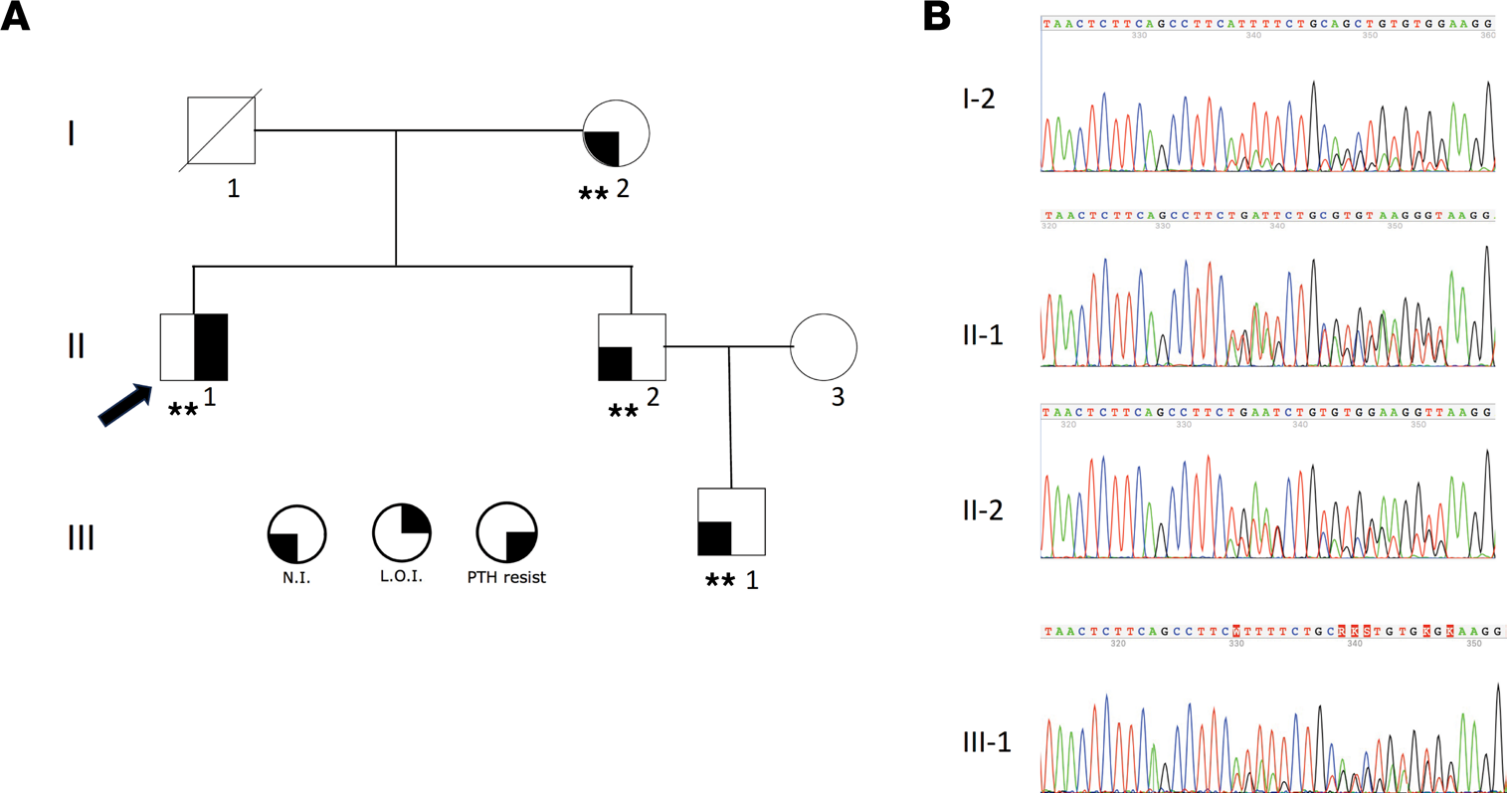
Pedigree of family 3 and the identified 6-bp deletion. (**A**) Pedigree of family 3 with AD-PHP1B. **Tested positive for the 6-bp deletion (20[GRCh37]:g.57419071–57419076delTTCATT) associated with a nearby single-nucleotide transversion (20[GRCh37]:g.57419082C>A) in *cis*. (**B**) Sanger sequencing in available family members of family 2. N.I., normal imprinting (methylation); L.O.I., loss of imprinting (abnormal methylation); PTH resist, resistance to parathyroid hormone.

**Figure 5:**
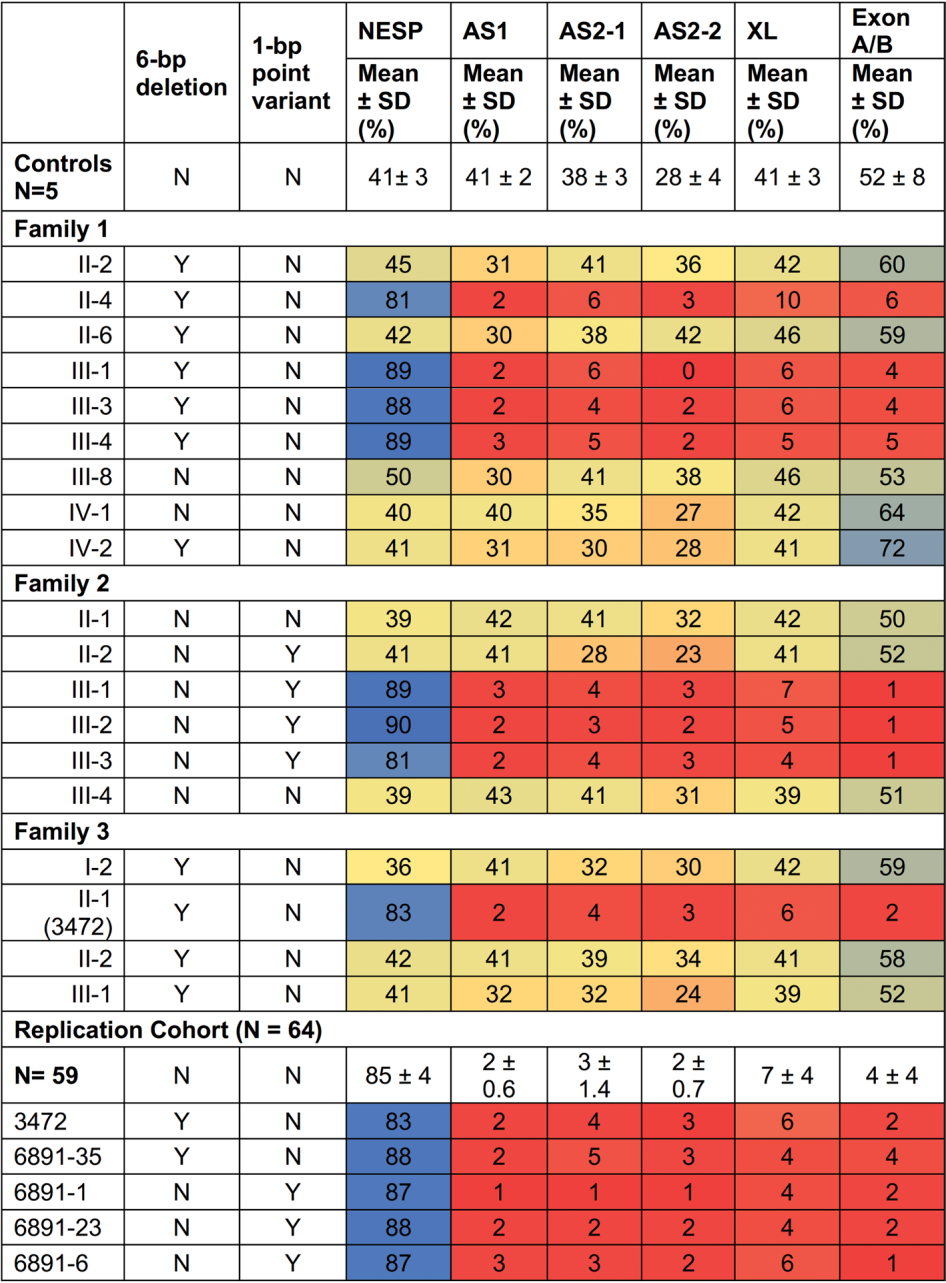
Methylation analyses. Colors were used to indicate methylation levels: red for marked reduction, blue for marked increase, and yellow for normal levels. Shaded red or blue represent moderate reductions or increases in methylation.

To further investigate the prevalence of the 2 genetic defects that we identified, we performed a replication study using an independent cohort of individuals with PHP1B. We performed Sanger sequencing on 64 additional unrelated individuals with PHP1B who were not previously reported in the literature and who have broad methylation defects (see above). We identified 2 additional patients, 3472 (II-1 in family 3, Figure 4A) and 6891-35 with apparent sporadic PHP1B, who carried the same complex 6-bp deletion (associated with the transversion in *cis*) as members of family 1 (Figure 4B and Figure 5), indicating that this small deletion is an uncommon but recurrent variant in PHP1B patients with global methylation defects. DNA was available from additional unaffected relatives of individual II-1 (family 3, Figure 4A) and Sanger sequencing demonstrated the same 6-bp deletion in his unaffected mother, brother, and nephew (Figure 4, A and B). DNA methylation analysis (Figure 5) showed that the affected individual (II-1) had a global defect in methylation that is consistent with inheritance of a defective *GNAS* allele from his unaffected mother (I-2), who had normal methylation status. By contrast, the proband’s brother, II-2, who had also inherited the defective *GNAS* allele from their mother, was unaffected and had normal methylation on *GNAS* DMRs. Finally, the proband’s unaffected nephew, individual III-1, also had normal methylation of the defective *GNAS* allele that he had inherited from his father, II-2. Therefore, the genetic and epigenetic findings in family 3 confirm and extend the findings in family 1 and further illustrate that maternal inheritance of this 6-bp deletion does not always lead to the methylation defects that cause PHP1B.

We also identified 3 additional unrelated patients, 6891-1, 6891-23, and 6891-6, with sporadic PHP1B, who carried the same point variant (20[GRCh37]:g.57,416,931G>A) in the first intronic region of the *NESP* transcript and near the donor splice site of *AS1* exon 4 as members of family 2 (Figure 5).

### Methylation analysis of DMRs.

We performed a comprehensive analysis of the methylation status of CpG sites within 6 regions that correspond to the 5 *GNAS* DMRs (Figure 1) for a control group of normal individuals, members of families 1 and 2, a replication cohort of patients with PHP1B who had global methylation defects, and members of family 3 who were related to the affected proband included in the replication cohort (Figure 5). DNA from healthy individuals showed 41%–52% methylation at DMRs corresponding to NESP, AS1, XL, and A/B. Remarkably, normal individuals showed moderate reductions in methylation at *GNAS-AS2-1*:TSS-DMR and *GNAS-AS2-2*:TSS-DMR, while individuals with sporadic PHP1B and global methylation defects showed marked reductions in methylation at these 2 sites*,* similar to findings reported by Hanna et al. (19). All patients in our study with sporadic PHP1B or AD-PHP1B had global methylation defects and showed increased methylation at *GNAS-NESP*:TSS-DMR and marked reductions in methylation at *GNAS-AS1*:TSS-DMR and both regions of the *GNAS-AS2-2*:TSS-DMR. By contrast, Hanna et al. (19) reported apparently increased methylation at *GNAS-AS2:*TSS-DMR in 3 affected members of an AD-PHP1B family who did not have an *STX16* deletion, but these individuals differed from those whom we studied as they did not have evidence for global methylation defects and showed normal methylation at *GNAS-NESP*:TSS-DMR*, GNAS-XL*:EX1-DMR, and *GNAS-AS1*:TSS*-*DMR.

### Quantification of GNAS transcript expression.

**Figure 6:**
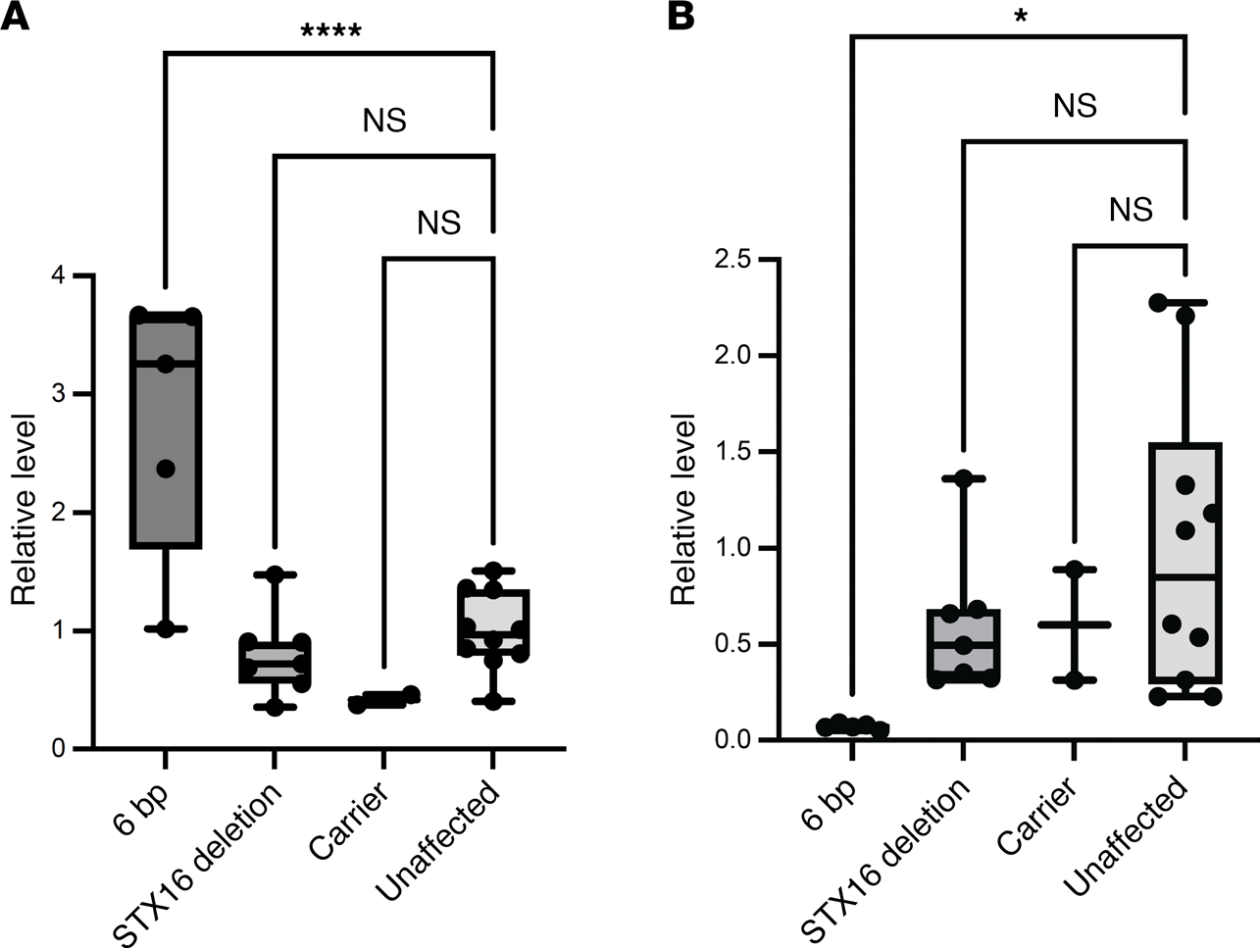
GNAS transcript expression. RT-qPCR showing higher levels of AS transcripts (**A**) and lower levels of the NESP transcripts (**B**) in patients with 6-bp deletion (*n* = 5) compared with patients with pathogenic STX16 deletion (*n* = 7), unaffected 6-bp deletion carriers (*n* = 2), or unrelated controls (*n* = 10). Values were normalized to 10 unaffected controls. Normalized values are illustrated by the box-and-whisker plot, where the center line represents the median, the box limits represent the interquartile range, and the whiskers represent the minimum to maximum data range. Comparisons between multiple groups were done by 1-way ANOVA followed by Dunnett’s test using GraphPad Prism. *P_adj_ < 0.05; ****P_adj_ < 0.0001.

We assessed the effect of the 6-bp deletion on transcription of AS and NESP by quantitative real-time reverse transcription PCR (RT-qPCR). These quantitative analyses showed that PHP1B individuals with the 6-bp deletion had significantly greater levels of the AS transcripts (*P_adj_* < 0.0001; Figure 6A) and lower levels of the NESP transcripts (*P_adj_* < 0.05; Figure 6B) in lymphoblastoid cell lines (LCLs) than did control normal individuals, which was consistent with the loss of imprinting at the AS promoter. By contrast, unaffected relatives in family 1 with the 6-bp deletion (carrier) or with wild-type *GNAS* alleles had normal levels of AS and NESP transcripts (Figure 6), consistent with normal methylation at the XL/AS DMR. Finally, LCLs from PHP1B patients with *STX16* deletions, who have normal methylation at the AS promoter, showed normal levels of AS and NESP transcripts.

## Discussion

PHP1B is now recognized as an epigenetic disorder in which expression of G_s_α from the maternal *GNAS* allele is disrupted. Several mechanisms have been identified as the cause of epigenetic diseases, including stable changes in DNA methylation, posttranslational histone modification, and/or production of noncoding RNA. These defects are termed epimutations when they are directly involved as the molecular basis of the disease and can be separated into 2 types, primary and secondary, the latter occurring as the result of a DNA variant that affects a *cis*- or *trans*-acting factor. The genetic basis for the aberrant methylation of DMRs in PHP1B appears to be heterogeneous. Recurrent deletions within the maternal *STX16* allele that include exon 4 and which disrupt a putative ICR termed STX16-ICR represent the predominant cause of AD-PHP1B and are associated with an imprinting defect that is limited to LOM at exon A/B (32). By contrast, the genetic basis for most cases of sporadic PHP1B, as well as some cases of AD-PHP1B, that are associated with more extensive imprinting defects affecting methylation at all DMRs has been more elusive. Rare maternally inherited heterozygous deletions involving the NESP55 exon and/or AS exons and introns have been identified in some PHP1B patients with broad methylation defects at the *GNAS* cluster (summarized in Figure 1), as these deletions presumably affect a primary ICR, termed NESP-ICR, that controls DMRs throughout the *GNAS* cluster (32). These observations have led to the notion that primary somatic epimutations rather than genetic defects account for the imprinting abnormalities in most PHP1B with broad methylation defects (28). Here we describe the application of GS to identify 2 small, intronic *GNAS* variants in patients with PHP1B who have extensive methylation defects. One variant is a complex 6-bp deletion (20[GRCh37]:g.57419071–57419076delTTCATT) associated with a nearby single-nucleotide transversion (20[GRCh37]:g.57419082C>A) that is intronic to both NESP55 and AS and is within the NESP-ICR. We found this variant on the maternal *GNAS* alleles of all affected individuals of 2 AD-PHP1B kindreds, families 1 and 3, as well as 1 individual with apparently sporadic PHP1B. Notably, this variant showed reduced penetrance in both familial cases, as not all individuals who carried the variant on a maternal *GNAS* allele manifested the imprinting defect that causes PHP1B. The identification of this variant in family 1 provides molecular confirmation of our previous linkage analysis that first showed a lack of concordance between aberrant methylation and a putative genetic defect within the maternal *GNAS* locus (42). A similar lack of co-segregation between aberrant methylation and a small deletion within the NESP-ICR of the maternal *GNAS* allele was subsequently reported in another PHP1B family with broad methylation defects (37). The incomplete penetrance of these small deletions may indicate that they are located near a boundary for the *cis*-acting element on the maternal *GNAS* allele that is required for methylation of the primary ICR for *GNAS*.

We also identified a point variant in affected members of the second multigenerational family with AD-PHP1B as well as 3 additional individuals with apparently sporadic PHP1B that is intronic to *AS2* and *AS3* exons. Therefore, this appears to be a second small variant that can cause broad methylation defects. In both cases, the identification of these variants in other, nonrelated patients with PHP1B suggests that the 2 changes are either recurrent pathogenic variants or very rare deleterious polymorphisms.

What can be the basis for the extensive imprinting defect within all *GNAS* DMRs, with GOM at the DMR for NESP55 and LOM at the DMRs for XL, AS1, and exon A/B, in these patients with such small mutations? Nearly all previously described PHP1B patients with broad methylation defects had larger deletions that included the NESP55 exon and/or AS exons on the maternal *GNAS* allele (28, 36–39) (Figure 1). By removing the unmethylated maternal NESP55 DMR, these deletions lead to an artifactual GOM at the DMR for NESP55 and disrupt normal transcription of NESP55, which is maternally expressed for approximately 80 kb across the entire *GNAS* locus. More importantly perhaps, it is likely that these deletions disrupt normal transcription of NESP55. Experimental studies have shown that transcription of NESP55 is required for proper methylation of downstream DMRs, as truncation (46, 47) or deletion (48) of *Nesp* in mice prevents acquisition of normal methylation at the downstream Nespas/Gnasxl and exon 1a DMRs in the oocyte. Therefore, deletions that include NESP55 and/or portions of AS exons lead to a broad methylation defect that has been termed H-L-L (or H-L-L-L), as shown in Figure 1. By contrast, discrete deletions that are limited to NESP55 (28, 49) result in a loss of imprinting that is limited to exon A/B (H-N-L; Figure 1), suggesting that transcription from the NESP55 promoter occurs but is prematurely terminated upstream of exon A/B.

The broad methylation defects in the PHP1B patients that we have studied are consistent with disruption of NESP55 transcription, but how can such small variants account for this profound defect in imprinting? Control of NESP55 transcription has been intensively studied over the past decade and important observations have demonstrated that transcription of AS regulates methylation of the NESP-ICR. The *GNAS* cluster contains 2 CpG islands with the characteristics of a germline ICR; these regions are differentially methylated in the germline and the differential methylation is maintained in the somatic tissues of the offspring. The principal ICR for the *GNAS* cluster contains the *AS* promoter (Figure 1A), and lies within the *AS* and *XL* DMR (50, 51); a second germline ICR is within the exon *A/B* DMR (52), and specifically controls maternal expression of *A/B* transcripts and the imprinted expression of transcripts encoding G_s_α. Importantly, the third DMR in the *GNAS* locus is the NESP-ICR, which is a somatic DMR that becomes methylated on the paternal allele after fertilization as a consequence of transcription of *AS* (50, 52, 53). By contrast, acquisition of CpG methylation within the maternal *AS* ICR during oogenesis prevents transcription of *AS* in somatic cells after fertilization and thereby assures that the *NESP* DMR is not methylated, with consequent transcription of *NESP* from the maternal allele. As we had LCLs from only patients with the 6-bp deletion, we were unable to investigate the effect of both mutations on *AS* and *NESP55* transcription. Nevertheless, our RT-qPCR data showed that transcription of *NESP55* was markedly reduced only in affected patients who had increased methylation of the NESP-ICR. Certainly, lack of NESP transcription provides a cogent explanation for the loss of methylation at the downstream DMRs. Surprisingly, our data also showed that transcription of *AS* was increased in these same patients, which could provide an explanation for methylation of the maternal NESP-ICR. Thus, we propose that the methylation status of the maternal *GNAS* DMRs in the patients we describe here is consistent with acquisition of NESP-ICR methylation as a result of abnormal activation of *AS* transcription. Taken together, these observations provide a putative mechanistic framework to explain the epigenetic changes observed in PHP1B patients who have genetic defects that disrupt transcription of *NESP55* upstream of exon A/B (33–35, 54) and align with the concept that DNA methylation is established in the oocyte in response to active transcription rather than by specific DNA sequence motifs or properties (55, 56). Thus, transcription initiating at the *NESP55* promoter and proceeding through the *GNAS* locus is required for acquisition of maternally methylated germline DMRs at XL/AS and exon A/B.

While these studies were in progress, Iwasaki et al. reported the application of human embryonal stem cells (hESCs) as a human cellular model to investigate the mechanistic basis for epigenetic defects in PHP1B in general and loss of methylation at the A/B DMR in particular (32). Specifically, using hESC clones with maternal NESP-ICR ablation, they showed that the NESP-ICR, and *NESP55* transcription, is required for methylation and transcriptional silencing of maternal A/B. However, it remains unexplained why this model failed to show hypomethylation at DMRs containing the AS and XL promoters, as occurs in PHP1B patients with defects in NESP55-ICR. In addition, they have proposed that the STX16-ICR behaves as a long-range enhancer of *NESP55* transcription based on experiments in which deletions within the STX16-ICR led to decreased transcription of NESP and consequent hypomethylation of maternal A/B (32). By contrast, our studies in LCLs from PHP1B patients with *STX16* deletions showed normal levels of *NESP55* transcription. The basis for these differences is uncertain, but may reflect variations in cell types (i.e., hESC versus LCL) or culture conditions. Nevertheless, it is still conceivable that the STX16-ICR functions as an enhancer of *NESP55* transcription, but that disruption of *STX16* leads to premature termination of *NESP55* transcription downstream of the AS and XL DMRs and thereby affects methylation only at the A/B DMR. Future studies will address these discrepant results that may reflect stage- and cell-specific differences between hESCs and LCLs.

In conclusion, our studies demonstrate the power of GS in identifying small, recurrent, genetic variants that are responsible for broad epigenetic defects in some patients with PHP1B. Surprisingly, we found that at least one of these genetic defects was incompletely penetrant, which can complicate clinical evaluation of inheritance patterns. Finally, our observation that relatively small variants downstream of *NESP55* can lead to derepression of *AS* transcription provides a new pathological basis for understanding aberrant methylation of the NESP-ICR and decreased transcription of *NESP55*.

## Methods

### Sex as a biological variable.

We studied both male and female individuals and similar findings are reported for both sexes.

### Study population.

We studied 2 families (see below) that included multiple members with AD-PHP1B and global methylation defects in *GNAS* DMRs. In addition, we included a replication cohort of 64 PHP1B individuals consisting of 55 patients with sporadic PHP1B and 9 probands with familial AD-PHP1B, all of whom had biochemical evidence for PTH resistance, with elevated serum levels of PTH and phosphate and low serum calcium levels, mild or no features of AHO, and in some cases mildly elevated serum TSH levels. All individuals had 2 wild-type copies of *GNAS* exons 1–13, global methylation defects, and lacked a causative genetic deletion in *STX16* or NESP55 or AS exons by exome sequencing. We extracted DNA from peripheral blood leukocytes or LCLs that were generated by EBV transformation of peripheral blood B lymphocytes using standard techniques (57).

### Family 1.

This kindred (Figure 2A) has been previously described as family R (42, 45) or F9 (28) in previous publications from our group. Affected individuals have evidence of PTH resistance (hypocalcemia or normocalcemia with elevated serum levels of intact PTH), normal thyroid function tests, and lack features of AHO. All affected individuals manifested a global defect in methylation that was linked to a presumed genetic defect in the *GNAS* locus (42, 45). Sanger sequencing of the 13 coding exons and exon/intron boundaries of the *GNAS* gene was normal and subsequent exome sequencing did not disclose a causative genetic mutation. Haplotype analyses showed unexpected discordance between the presumed genetic defect in or near *GNAS* and the methylation defect (i.e., not all individuals who carried the proposed disease-associated haplotype manifested the broad methylation defect or biochemical evidence of PTH resistance) (42, 43), leading to the conclusion that the genetic defect was incompletely penetrant.

### Family 2.

This kindred (Figure 3A) has been described as family F6 in a previous publication from our group (28). The proband (F6-4; III-1) is a female with a history of mild developmental delays and primary hypothyroidism with onset in early childhood. She presented at age 6 years with a generalized seizure, at which time her serum calcium was 5.7 mg/dL, serum phosphate was 8.8 mg/dL, and serum intact PTH was marked elevated at 286 pg/mL. She improved quickly with calcium and calcitriol treatment, in addition to continued levothyroxine, and was discharged to home. Her past history revealed prior concerns for Beckwith-Wiedemann syndrome (BWS) shortly after birth when pharyngomalacia and macroglossia were identified during evaluation for poor feeding and irritability. Although genetic testing was negative, she was subsequently monitored by medical genetics for her first 6 years of life for possible BWS due to clinical features, including macroglossia, an umbilical hernia, and a facial nevus simplex.

She has a round face with a normal appearing tongue and bilateral brachydactyly E manifest as short 4th metacarpals and 4th and 5th toes. She had continued to have trouble with motor incoordination and learning, requiring an individualized education plan but she was able to attend regular education classes. She had spontaneous menarche at 11 years of age and is of normal stature (CDC height in 42nd percentile at age 15).

The proband has 3 younger siblings who are dizygotic triplets, an unaffected female (III-4; F6-1) and 2 affected monozygotic males (III-2, F6-5 and III-3, F6-6). Her twin younger brothers, now aged 10 years, were found to have primary hypothyroidism and elevated serum levels of phosphate and intact PTH, but normal calcium levels. Both affected brothers lack brachydactyly but have mild developmental delays, and round faces. The father (II-1; F6-2) and mother (II-2; F6-3) are non-consanguineous and have normal physical appearances and physiologic mineral metabolism. A commercial next-generation sequencing panel for genes associated with hypoparathyroidism was negative, but methylation studies of exon A/B at Johns Hopkins Clinical Genetics laboratory showed loss of methylation for affected individuals III-1, III-2, and III-3 (all approximately 3%, normal ≥43%) thereby confirming a diagnosis of PHP1B. The proband’s unaffected mother had normal methylation of exon A/B (48%). A maternal male cousin (III-5) was subsequently diagnosed with PTH resistance and likely PHP1B based on loss of methylation at exon A/B of the *GNAS* gene (5%). His mother (II-5) is unaffected, but has not undergone molecular testing.

Given the diagnosis of AD-PHP1B, additional genetic studies were performed. *STX16* sequencing and deletion duplication analysis were normal and clinical exome sequencing of the proband and both parents with sibling controls did not reveal a causative mutation.

### Family 3.

The proband (II-1, Figure 4A) was included within our replication cohort (3472, see below). He had been described as individual S26 (28) and 13 (58) with sporadic PHP1B in previous publications from our group. He had been referred to our clinic as an adult patient with PHP1A based on short stature, brachydactyly type E, cognitive delay, and resistance to PTH and TSH. There were no subcutaneous ossifications. When first evaluated in our clinic, he was receiving dihydrotachysterol for PTH resistance and levothyroxine for mild hypothyroidism (pretreatment data during childhood were not available). Sanger sequencing of exons 1–13 of the *GNAS* gene was normal. His biochemical evaluation while on treatment showed normal serum levels of calcium, phosphorus, PTH and TSH, as well as normal serum concentrations of luteinizing hormone, follicle-stimulating hormone, and testosterone; he had normal growth hormone reserve (58). Subsequent exome sequencing was normal and methylation analyses revealed a broad methylation defect in *GNAS* DMRs that confirmed a diagnosis of PHP1B. His parents, a brother, and a nephew (Figure 4A) were all unaffected and had normal serum levels of PTH, calcium, phosphorus, and TSH (data not shown).

### Methylation analysis of GNAS DMRs.

Methylation analysis of *GNAS* DMRs (Figure 1A) was determined as previously described (28, 59) by PCR amplification of bisulphite-modified, RNase-treated blood genomic DNA followed by pyrosequencing using assays designed in collaboration with EpigenDx. Nucleotide sequences in *GNAS* (chr20q13.32) that were analyzed were in the DMRs as noted (positions per GRCh37/hg19): NESP55 (ADS471: g.57,415,807–57,415,853), XL (ADS470: g.57,429,235–57,429,362), exon A/B (ADS464: g.57,464,773–57,464,927), AS1 (ADS3559-FS: g.57,426,926–57,426,950), AS2-1 (ADS3560-FS: g.57,427,692–57,427,762), and AS2-2 (ADS3563-FS and ADS3562-FS: g.57,427,813–57,427,869 and g.57,427,942–57,427,994, respectively). The assay for AS1 is confounded by the presence of a C>T polymorphism at chr20:57426935 (https://gnomad.broadinstitute.org/variant/20-57426935-C-T?dataset=gnomad_r2_1) that has a MAF of 11%. The assays for AS1 and the two AS2 DMRs were based on primer sequences and experimental conditions as described by Hanna et al. (19). Bisulfite pyrosequencing is a sequencing-by-synthesis method used to quantitatively determine the methylation of individual cytosines from PCR amplicons of a region up to 115 bases in length. Methylation is expressed as the percentage of cytosines within the CpG sequences that are methylated.

### GS and bioinformatic analysis.

We performed GS with paired-end 100-bp reads (59). Libraries were generated from genomic DNA using the Illumina TruSeq DNA PCR-Free Library Prep Kit. All the raw reads were aligned to the reference human genome using the Burrows-Wheeler Aligner (BWA-Mem; ref. 60) and SNVs and small insertions/deletions (indels) were captured using the Genome Analysis Tool Kit (GATK; ref. 61). ANNOVAR (62) and SnpEff (63) were subsequently used to functionally annotate the variants. BAM files generated by GATK were fed to multiple short-read structural variant callers, including BreakDancer (64), Manta (65), Wham (66), and CNVnator (67), to capture SVs with default parameters. Similarly, the split and discordant reads files were generated by SpeedSeq (68) and were provided as inputs to Lumpy (69), another SV calling program. The confirmatory genotyping of *GNAS* intronic variants was performed by Sanger sequencing by using primers 5′-GGAGGAGGAGCAGGAGAATA-3′ and 5′-CAGTTGAGCCAGCACATGAC-3′ (658 bp), and 5′-ATGGTCACGTCGGGGTATTG-3′ and 5′-CCTCCTTTTCGACGACTGATC-3′ (288 bp).

### Quantification of relative levels of GNAS transcripts.

Total RNA was extracted from LCLs by the RNase-Free DNase Sets and RNeasy Mini Kits (all from QIAGEN), according to the manufacturer’s instructions. RNA concentrations and purity were assessed with the NanoDrop 1000 spectrophotometer (Thermo Fisher Scientific), and RNA integrity was evaluated with the Bioanalyzer 2100 (Agilent Technologies). cDNA was synthesized using SuperScript III First-Strand Synthesis SuperMix (Invitrogen/Thermo Fisher Scientific). We performed RT-qPCR using the Quantitect SYBR Green PCR kit (QIAGEN) and the data were analyzed by using the ΔΔCt method. We used AS-specific primers M (5′-GGTTTTTCAGAGTCTGGTAGCC-3′) and N (5′-GAGGAGCAAGAAGATTTCCA-3′) to amplify AS transcripts (37) and NESP-specific forward primer (5′-AAGAGTCGAAGGAGCCCAAGGAG-3′) with a reverse primer (5′-CCATTAAACCCATTAACATGCAG-3′) located in *GNAS* exon 2 to amplify NESP transcripts (32). We normalized expression of AS and NESP transcripts to the level of expression of a reference gene by measuring ΔΔCt values determined by RT-qPCR of β-glucuronidase (*GUSP*) transcripts in the same cDNA samples and expressed values as a percentage of control based on results of 10 individuals with normal *GNAS* genes. Duplicate sets of samples were produced with RT omitted to detect amplification from contaminating DNA.

### Statistics.

Pyrosequencing results are presented as the mean ± SD of methylation in *GNAS* DMRs in DNA from the normal control group. Data from RT-qPCR experiments were analyzed by first calculating the mean of 3 replicates for each sample and then performing 1-way ANOVA followed by Dunnett’s multiple-comparison test. *P* values of less than 0.05 were considered significant. GraphPad Prism software was used for the statistical analyses.

### Study approval.

The protocol was approved by the Children’s Hospital of Philadelphia institutional review board and informed consent/assent was obtained as appropriate.

### Data availability.

Supporting data for full values underlying the data presented in the graphs are provided in the supplemental Supporting Data Values file.

## Author contributions

DL designed research studies, conducted experiments, acquired data, analyzed data, and wrote the manuscript. SJB designed the research studies and wrote the manuscript. CH and HS conducted experiments and acquired data. MRZR acquired data, analyzed data, and wrote the manuscript. GRC designed research studies, analyzed data, and wrote the manuscript. MBS conducted experiments, acquired data, and wrote the manuscript. MAL designed the research studies, acquired data, analyzed data, and wrote the manuscript.

## Supplementary Material

Supporting data values

## Version 1 11/14/2024

In-Press Preview

## Version 2 12/20/2024

Electronic publication

## References

[1] Thiele S (2016). From pseudohypoparathyroidism to inactivating PTH/PTHrP signalling disorder (iPPSD), a novel classification proposed by the EuroPHP network. Eur J Endocrinol.

[2] Mantovani G (2018). Diagnosis and management of pseudohypoparathyroidism and related disorders: first international consensus statement. Nat Rev Endocrinol.

[3] Mantovani G (2020). Recommendations for diagnosis and treatment of pseudohypoparathyroidism and related disorders: an updated practical tool for physicians and patients. Horm Res Paediatr.

[4] Linglart A (2018). Pseudohypoparathyroidism. Endocrinol Metab Clin North Am.

[5] Levine MA (1991). Mapping of the gene encoding the alpha subunit of the stimulatory G protein of adenylyl cyclase (GNAS1) to 20q13.2----q13.3 in human by in situ hybridization. Genomics.

[6] Hayward BE (1998). The human GNAS1 gene is imprinted and encodes distinct paternally and biallelically expressed G proteins. Proc Natl Acad Sci U S A.

[7] Hayward BE (1998). Bidirectional imprinting of a single gene: GNAS1 encodes maternally, paternally, and biallelically derived proteins. Proc Natl Acad Sci U S A.

[8] Peters J (1999). A cluster of oppositely imprinted transcripts at the Gnas locus in the distal imprinting region of mouse chromosome 2. Proc Natl Acad Sci U S A.

[9] Chen M (2009). Central nervous system imprinting of the G protein G(s)alpha and its role in metabolic regulation. Cell Metab.

[10] Klenke S (2011). A novel aspect of GNAS imprinting: higher maternal expression of Gαs in human lymphoblasts, peripheral blood mononuclear cells, mammary adipose tissue, and heart. Mol Cell Endocrinol.

[11] Liu J (2003). The stimulatory G protein alpha-subunit Gs alpha is imprinted in human thyroid glands: implications for thyroid function in pseudohypoparathyroidism types 1A and 1B. J Clin Endocrinol Metab.

[12] Mantovani G (2002). The gsalpha gene: predominant maternal origin of transcription in human thyroid gland and gonads. J Clin Endocrinol Metab.

[13] Mantovani G (2004). Biallelic expression of the Gsalpha gene in human bone and adipose tissue. J Clin Endocrinol Metab.

[14] Zheng H (2001). Galphas transcripts are biallelically expressed in the human kidney cortex: implications for pseudohypoparathyroidism type 1b. J Clin Endocrinol Metab.

[15] Weinstein LS (2000). Variable imprinting of the heterotrimeric G protein G(s) alpha-subunit within different segments of the nephron. Am J Physiol Renal Physiol.

[16] Crane JL (2009). Imprinting status of Galpha(s), NESP55, and XLalphas in cell cultures derived from human embryonic germ cells: GNAS imprinting in human embryonic germ cells. Clin Transl Sci.

[17] Germain-Lee EL (2002). Paternal imprinting of Galpha(s) in the human thyroid as the basis of TSH resistance in pseudohypoparathyroidism type 1a. Biochem Biophys Res Commun.

[18] Rochtus A (2016). Genome-wide DNA methylation analysis of pseudohypoparathyroidism patients with GNAS imprinting defects. Clin Epigenetics.

[19] Hanna P (2021). A novel familial PHP1B variant with incomplete loss of methylation at GNAS-A/B and enhanced methylation at GNAS-AS2. J Clin Endocrinol Metab.

[20] Levine MA (1988). Genetic deficiency of the alpha subunit of the guanine nucleotide-binding protein Gs as the molecular basis for Albright hereditary osteodystrophy. Proc Natl Acad Sci U S A.

[21] Patten JL (1990). Mutation in the gene encoding the stimulatory G protein of adenylate cyclase in Albright’s hereditary osteodystrophy. N Engl J Med.

[22] Bastepe M (2001). Paternal uniparental isodisomy of chromosome 20q--and the resulting changes in GNAS1 methylation--as a plausible cause of pseudohypoparathyroidism. Am J Hum Genet.

[23] Fernandez-Rebollo E (2010). New mechanisms involved in paternal 20q disomy associated with pseudohypoparathyroidism. Eur J Endocrinol.

[24] Dixit A (2013). Pseudohypoparathyroidism type 1b due to paternal uniparental disomy of chromosome 20q. J Clin Endocrinol Metab.

[25] Takatani R (2015). Similar frequency of paternal uniparental disomy involving chromosome 20q (patUPD20q) in Japanese and Caucasian patients affected by sporadic pseudohypoparathyroidism type Ib (sporPHP1B). Bone.

[26] Bastepe M (2011). Paternal uniparental isodisomy of the entire chromosome 20 as a molecular cause of pseudohypoparathyroidism type Ib (PHP-Ib). Bone.

[27] Colson C (2019). High frequency of paternal iso or heterodisomy at chromosome 20 associated with sporadic pseudohypoparathyroidism 1B. Bone.

[28] Danzig J (2021). High-throughput molecular analysis of pseudohypoparathyroidism 1b patients reveals novel genetic and epigenetic defects. J Clin Endocrinol Metab.

[29] Milioto A (2022). Lack of GNAS remethylation during oogenesis may be a cause of sporadic pseudohypoparathyroidism type Ib. J Clin Endocrinol Metab.

[30] Bastepe M (2003). Autosomal dominant pseudohypoparathyroidism type Ib is associated with a heterozygous microdeletion that likely disrupts a putative imprinting control element of GNAS. J Clin Invest.

[31] Linglart A (2005). A novel STX16 deletion in autosomal dominant pseudohypoparathyroidism type Ib redefines the boundaries of a cis-acting imprinting control element of GNAS. Am J Hum Genet.

[32] Iwasaki Y (2023). The long-range interaction between two GNAS imprinting control regions delineates pseudohypoparathyroidism type 1B pathogenesis. J Clin Invest.

[33] Grigelioniene G (2017). A large inversion involving GNAS exon A/B and all exons encoding Gsα is associated with autosomal dominant pseudohypoparathyroidism type Ib (PHP1B). J Bone Miner Res.

[34] Miller DE (2022). Targeted long-read sequencing identifies a retrotransposon insertion as a cause of altered GNAS exon A/B methylation in a family with autosomal dominant pseudohypoparathyroidism type 1b (PHP1B). J Bone Miner Res.

[35] Kawashima S (2022). Familial pseudohypoparathyroidism type IB associated with an SVA retrotransposon insertion in the GNAS locus. J Bone Miner Res.

[36] Bastepe M (2005). Deletion of the NESP55 differentially methylated region causes loss of maternal GNAS imprints and pseudohypoparathyroidism type Ib. Nat Genet.

[37] Chillambhi S (2010). Deletion of the noncoding GNAS antisense transcript causes pseudohypoparathyroidism type Ib and biparental defects of GNAS methylation in cis. J Clin Endocrinol Metab.

[38] Rezwan FI (2015). Very small deletions within the NESP55 gene in pseudohypoparathyroidism type 1b. Eur J Hum Genet.

[39] Takatani R (2016). Analysis of multiple families with single individuals affected by pseudohypoparathyroidism type Ib (PHP1B) reveals only one novel maternally inherited GNAS deletion. J Bone Miner Res.

[40] Perez-Nanclares G (2015). Pseudohypoparathyroidism type Ib associated with novel duplications in the GNAS locus. PLoS One.

[41] Nakamura A (2016). Complex genomic rearrangement within the GNAS region associated with familial pseudohypoparathyroidism type 1b. J Clin Endocrinol Metab.

[42] Jan de Beur S (2003). Discordance between genetic and epigenetic defects in pseudohypoparathyroidism type 1b revealed by inconsistent loss of maternal imprinting at GNAS1. Am J Hum Genet.

[43] Jan De Beur SM (2003). The pseudohypoparathyroidism type lb locus is linked to a region including GNAS1 at 20q13.3. J Bone Miner Res.

[44] Efstratiadis A (1980). The structure and evolution of the human beta-globin gene family. Cell.

[45] Jan de Beur SM (2000). Pseudohypoparathyroidism 1b: exclusion of parathyroid hormone and its receptors as candidate disease genes. J Clin Endocrinol Metab.

[46] Chotalia M (2009). Transcription is required for establishment of germline methylation marks at imprinted genes. Genes Dev.

[47] Williamson CM (2006). Identification of an imprinting control region affecting the expression of all transcripts in the Gnas cluster. Nat Genet.

[48] Frolich LF (2010). Targeted deletion of the Nesp55 DMR defines another Gnas imprinting control region and provides a mouse model of autosomal dominant PHP-Ib. Proc Natl Acad Sci U S A.

[49] Richard N (2012). A new deletion ablating NESP55 causes loss of maternal imprint of A/B GNAS and autosomal dominant pseudohypoparathyroidism type Ib. J Clin Endocrinol Metab.

[50] Coombes C (2003). Epigenetic properties and identification of an imprint mark in the Nesp-Gnasxl domain of the mouse Gnas imprinted locus. Mol Cell Biol.

[51] Williamson CM (2004). A cis-acting control region is required exclusively for the tissue-specific imprinting of Gnas. Nat Genet.

[52] Liu J (2000). Identification of a methylation imprint mark within the mouse Gnas locus. Mol Cell Biol.

[53] Grybek V (2014). Methylation and transcripts expression at the imprinted GNAS locus in human embryonic and induced pluripotent stem cells and their derivatives. Stem Cell Reports.

[54] Reyes M (2021). A novel GNAS duplication associated with loss-of-methylation restricted to exon A/B causes pseudohypoparathyroidism type Ib (PHP1B). J Bone Miner Res.

[55] Sendzikaite G, Kelsey G (2019). The role and mechanisms of DNA methylation in the oocyte. Essays Biochem.

[56] Ivanova E (2020). DNA methylation changes during preimplantation development reveal inter-species differences and reprogramming events at imprinted genes. Clin Epigenetics.

[57] Neitzel H (1986). A routine method for the establishment of permanent growing lymphoblastoid cell lines. Hum Genet.

[58] Germain-Lee EL (2003). Growth hormone deficiency in pseudohypoparathyroidism type 1a: another manifestation of multihormone resistance. J Clin Endocrinol Metab.

[59] Li D (2020). Intragenic deletions of GNAS in pseudohypoparathyroidism type 1A identify a new region affecting methylation of exon A/B. J Clin Endocrinol Metab.

[60] Li H, Durbin R (2009). Fast and accurate short read alignment with Burrows-Wheeler transform. Bioinformatics.

[61] DePristo MA (2011). A framework for variation discovery and genotyping using next-generation DNA sequencing data. Nat Genet.

[62] Wang K (2010). ANNOVAR: functional annotation of genetic variants from high-throughput sequencing data. Nucleic Acids Res.

[63] Cingolani P (2012). A program for annotating and predicting the effects of single nucleotide polymorphisms, SnpEff: SNPs in the genome of Drosophila melanogaster strain w1118; iso-2; iso-3. Fly (Austin).

[64] Chen K (2009). BreakDancer: an algorithm for high-resolution mapping of genomic structural variation. Nat Methods.

[65] Chen X (2016). Manta: rapid detection of structural variants and indels for germline and cancer sequencing applications. Bioinformatics.

[66] Kronenberg ZN (2015). Wham: identifying structural variants of biological consequence. PLoS Comput Biol.

[67] Abyzov A (2011). CNVnator: an approach to discover, genotype, and characterize typical and atypical CNVs from family and population genome sequencing. Genome Res.

[68] Chiang C (2015). SpeedSeq: ultra-fast personal genome analysis and interpretation. Nat Methods.

[69] Layer RM (2014). LUMPY: a probabilistic framework for structural variant discovery. Genome Biol.

